# Draft Genome Sequence of *Bifidobacterium animalis* subsp. *lactis* Strain CECT 8145, Able To Improve Metabolic Syndrome *In Vivo*

**DOI:** 10.1128/genomeA.00183-14

**Published:** 2014-03-27

**Authors:** E. Chenoll, F. M. Codoñer, A. Silva, J. F. Martinez-Blanch, P. Martorell, D. Ramón, S. Genovés

**Affiliations:** a Department of Food Biotechnology, Biópolis SL, Parc Científic Universitat de València, Paterna, Valencia, Spain; b Lifesequencing SL, Parc Científic Universitat de València, Paterna, Valencia, Spain

## Abstract

*Bifidobacterium animalis* subsp. *lactis* strain CECT 8145 is able to reduce body fat content and improve metabolic syndrome biomarkers. Here, we report the draft genome sequence of this strain, which may provide insights into its safety status and functional role.

## GENOME ANNOUNCEMENT

Bifidobacteria are common inhabitants of the human gut and play a significant role in establishing a well-balanced intestinal microbiota. Many authors have pointed to the relationship between gut microbiota and body weight regulation and, consequently, the influence of gut microbiota on obesity-related diseases (1, 2). The strain *Bifidobacterium animalis* subsp. *lactis* CECT 8145 has been proven to significantly reduce body fat content compared with other commercial strains (our unpublished data) in a *Caenorhabditis elegans* model (3, 4). Transcriptomic and metabolomic studies have enabled us to identify target genes in *C. elegans* that are differentially expressed after the ingestion of strain CECT 8145, thus indicating that the biological activities are directly related with its intake. Furthermore, a preclinical study carried out in obese Zücker rats showed a positive weight reduction effect and an improvement in biochemical parameters in the obese rats treated with probiotic CECT 8145 (our unpublished data).

In order to study the strain *B. animalis* subsp. *lactis* CECT 8145 in depth with regard to safety for human consumption, whole-genome sequencing was performed according to European Food Safety Authority (EFSA) and FAO/WHO recommendations. A paired-end sequencing strategy was adopted according to the standard protocol developed by 454 Life Sciences/Roche (GS FLX Titanium; 454 Life Sciences/Roche, Branford, CT). Initially, the pyrosequencing run rendered 434,581 reads, with an average read length of 319.63 nucleotides, totaling 138.9 Mb of sequencing throughput. High-quality sequences were assembled with the Newbler assembler version 2.6 (Life Sciences/Roche) using the default parameters. The assembly resulted in 99 contigs, with 31 of them >500 nucleotides. The *N*_50_ of the contig assembly is 149,259 nucleotides, and the longest is 322,919 nucleotides. Most of these contigs are arranged in five scaffolds. The *N*_50_ of the scaffolding and the largest scaffold is 1,923,368 nucleotides. The uncompleted draft genome of this strain is around 2.1 Mb in size (with a G+C content around 60.46%), and no plasmids have been detected.

Open reading frames (ORFs) were predicted using Glimmer version 3.02 (5–7). A total of 1,632 coding sequences and 56 RNAs were predicted and annotated (including four copies of rRNA genes and 52 predicted tRNAs). A nonmultiple of 3 detected in the rRNA indicates that there are ≥2 rRNA cassettes.

The genes associated with antibiotic resistance and putative nonspecific virulence factors are found in many *Bifidobacterium* genomes, including those of *B. animalis* species and other *Bifidobacterium* commercial strains. All the genes involved in antibiotic resistance are mainly associated with drug transport, and previous phenotypic results have shown that MIC values are below the EFSA breaking points (data not shown) while the putative virulence genes are common to commercial *Bifidobacterium* species.

In conclusion, the safety results obtained are very similar to those for other previously sequenced *B. animalis* and commercial *Bifidobacterium* strains; furthermore, the results obtained for *in vivo* studies show that *B. animalis* subsp. *lactis* CECT 8145 can be considered generally recognized as safe (GRAS)/qualified presumption of safety (QPS) (data not shown).

### Nucleotide sequence accession numbers.

This draft genome project has been deposited at DDBJ/EMBL/GenBank under the accession no. CBWX000000000. The version described in this paper is the first version, CBWX010000000.
